# Granger causality connectivity analysis of persistent atrial fibrillation dynamics reveals posterior wall mechanistic insights

**DOI:** 10.1016/j.hroo.2025.05.002

**Published:** 2025-05-11

**Authors:** Joseph Barker, Arunashis Sau, Nikesh Bajaj, Alex Jenkins, Alex Sharp, Xili Shi, Xinyang Li, Nabeela Karim, Balvinder Handa, Richard Chambers, Timothy Betts, Nicholas S. Peters, Tom Wong, Fu Siong Ng

**Affiliations:** 1 National Heart and Lung Institute, Imperial College London, London, United Kingdom; 2 Department of Cardiology, Imperial College Healthcare NHS Trust, London, United Kingdom; 3 Department of Engineering Science, Institute of Biomechanical Engineering, University of Oxford, Oxford, United Kingdom; 4 Department of Cardiology, Royal Brompton & Harefield Hospitals, Guy’s and St. Thomas’ NHS Foundation Trust, London, United Kingdom; 5 Acutus Medical, Carlsbad, California; 6 ChamberTech LTD, London Institute for Healthcare Engineering, London, United Kingdom; 7 Division of Cardiovascular Medicine, University of Oxford, Oxford, United Kingdom; 8 Department of Cardiology, Chelsea and Westminster Hospital NHS Foundation Trust, London, United Kingdom

**Keywords:** Atrial fibrillation, Granger causality, Posterior wall isolation, Pulmonary vein isolation, Mechanism

## Abstract

**Background:**

Adjunctive posterior wall isolation (PWI) to pulmonary vein isolation (PVI) has not demonstrated convincing benefit during atrial fibrillation (AF) ablation. To provide mechanistic insight for null PWI trials, we undertook Granger causality (GC) analysis of noncontact left atrial (LA) electroanatomic maps.

**Objective:**

This study aimed to apply GC to intracardiac electrograms to uncover patient-specific AF dynamics and describe a proof-of-concept approach to targeted PWI after PVI.

**Methods:**

A prospective cohort study was undertaken at Royal Brompton Hospital. Consecutive patients undergoing PVI with noncontact mapping (AcQmap; Acutus Medical) before and after PVI were included.

**Results:**

In 21 patients, causality pairing index, a GC measure of organization, was unchanged after PVI (overall, 0.087 ± 0.012 vs 0.086 ± 0.015; *P* = .64) or by region (posterior wall [PW], 0.084 ± 0.020 vs 0.079 ± 0.017; *P* = .20; rest of LA, 0.087 ± 0.013 vs 0.086 ± 0.016; *P* = .80). Directional dispersion, quantifying conduction heterogeneity, was lower in the PW than the rest of the LA (0.093 ± 0.036 vs 0.11 ± 0.043; *P* = .017) and increased after PVI (0.093 ± 0.036 vs 0.12 ± 0.043; *P* = .045), whereas there was no change in the rest of the LA (0.11 ± 0.034 vs 0.11 ± 0.030; *P* = .52). PW net outflow overall decreased after PVI (before, −0.0086 ± 0.047 vs −0.033 ± 0.054; *P* = .011) with a minority of patients exhibiting a net positive outflow from the PW.

**Conclusion:**

GC provides mechanistic insight into the null trials for PWI and identifies a minority of patients who may benefit. GC is positioned as a clinical decision tool to guide personalized persistent AF ablation strategies.

## Graphical abstract

**Figure:**
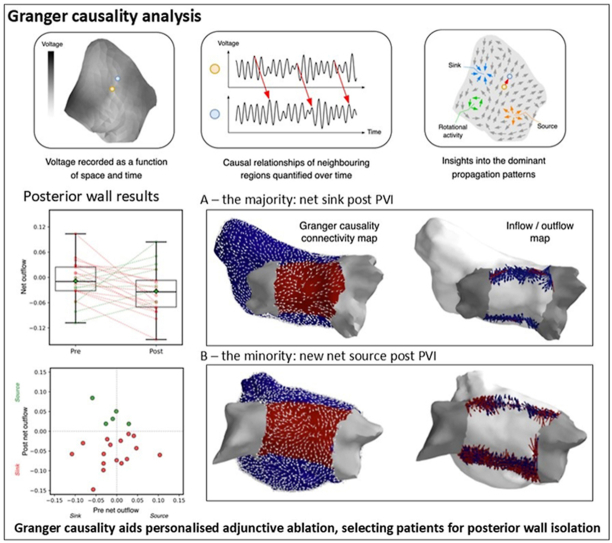

**Key Findings**

▪ Granger Causality (GC) analysis applied to non-contact atrial mapping reveals patient-specific AF propagation patterns and connectivity.
▪ Following pulmonary vein isolation (PVI), the left atrial posterior wall (PW) typically becomes a net sink, suggesting it is less likely to act as an AF driver, and explaining the lack of positive trials in this space.
▪ Directional dispersion—a measure of conduction heterogeneity—increases in the posterior wall post-PVI, indicating altered fibrillatory dynamics.
▪ Only a minority of patients demonstrated the PW as a net source post-PVI, suggesting that posterior wall isolation (PWI) may benefit only select individuals.
▪ GC analysis may serve as a valuable clinical decision tool to identify patients most likely to benefit from adjunctive PWI, potentially enabling personalized AF ablation strategies.

## Introduction

Pulmonary vein isolation (PVI) has been the mainstay of ablation for atrial fibrillation (AF) since its seminal description in 1998.^1^ It works by isolating the atria from pulmonary vein ectopy and may result in atrial substrate modification.^2^, ^3^, ^4^ PVI for paroxysmal AF has good success rates (> 70%) whereas PVI for persistent AF (PsAF) has more modest success rates with a ceiling of approximately 50%.^4^, ^5^, ^6^

Many adjunctive lesion sets have been studied to improve success of PsAF ablation; however, none have demonstrated convincing additional benefit beyond PVI alone.^6^ Posterior wall isolation (PWI), designed to isolate posterior wall (PW) sources of ectopy, initially showed promise in the literature. However, subsequent studies failed to demonstrate a clear benefit, with the recent results of the rigorously conducted randomized controlled CAPLA study, implementing both lesion-specific quality targets and intense/complete follow-up, showing no benefit of PWI + PVI vs PVI alone.^7^, ^8^, ^9^, ^10^, ^11^ Despite this, PWI remains in the AF catheter ablation consensus statements worldwide.^12^^,^^13^

We previously adapted Granger causality (GC) analysis, an econometric tool, for use in AF mapping.^14^ This method analyses the “causal” relationships between AF signals to build a picture of AF connectivity, providing insight into patient-specific AF dynamics. In this study, we performed GC analysis on AF mapping data before and after PVI, to better understand the role of the left atrial (LA) PW in AF pathophysiology and to provide some mechanistic underpinnings for the CAPLA study and more broadly the negative results of PWI randomized controlled trials.^6^

## Methods

### Ethical approval

This study was approved by the national ethics committee (National Health Service Health Research Authority). A written individual informed patient consent was obtained.

### Clinical data collection

A prospective cohort study was undertaken at Royal Brompton Hospital as previously described.^15^ Briefly, consecutive patients with PsAF undergoing AF ablation had LA electroanatomic maps collected using the AcQMap noncontact mapping catheter. AcQmap uses ultrasound to reconstruct 3-dimensional endocardial anatomy, then creates dipole density maps from unipolar electrograms, allowing for real-time, precise mapping of global cardiac electrical activity.^16^ The reconstructed unipolar electrograms were used in this analysis. AF data were analyzed before and after PVI but before any adjunctive ablations.^17^ The follow-up period lasted 12 months. Any instance of AF lasting more than 30 seconds, as identified through electrocardiograms or continuous heart rhythm monitoring, was classified as arrhythmia recurrence. Reports of arrhythmia recurrence were collected through a coordinated research and clinical network, which included dedicated patient phone lines and e-mails, reports from community primary care physicians, input from arrhythmia specialist nurses, and periodic outpatient reviews in secondary or tertiary care settings. Statistical analysis was undertaken on a complete case basis, with this manuscript prepared according to Strengthening the Reporting of Observational Studies in Epidemiology guidelines.^18^

### GC analysis

**Figure 1:**
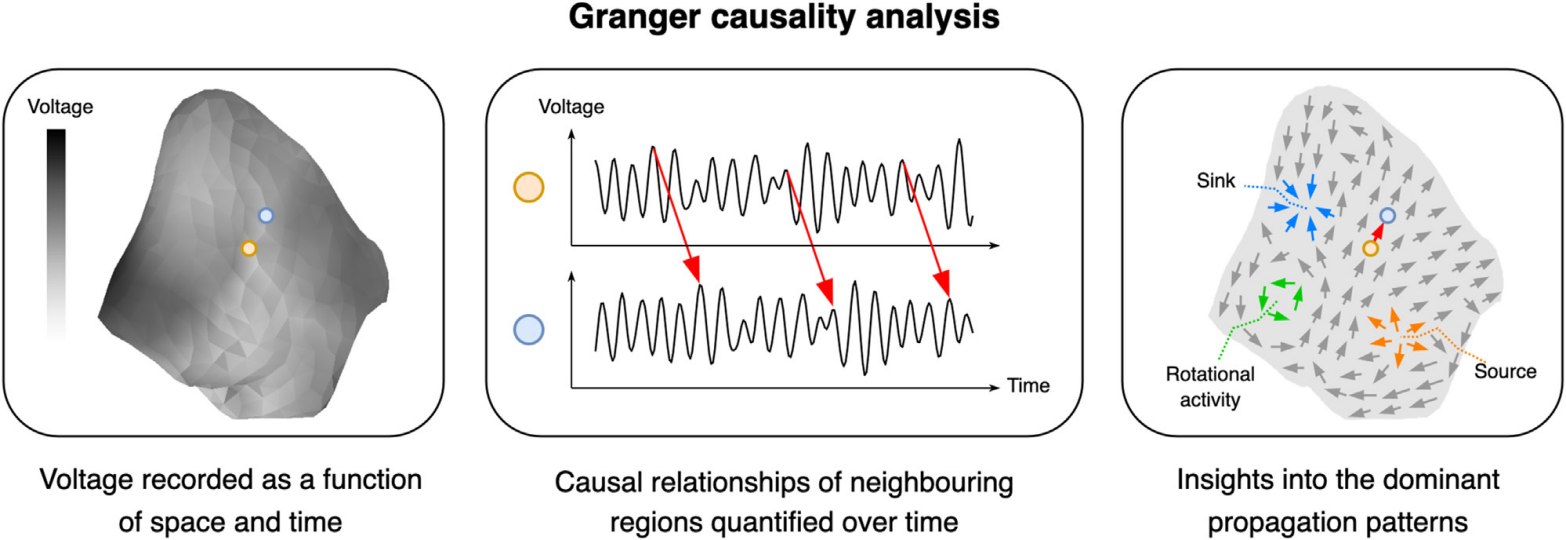
An overview of Granger causality analysis applied to simultaneous electroanatomic noncontact mapping.

GC was initially described as an econometric tool to quantify causal dependence between time series.^19^ We previously described the adaptation and application of GC to PsAF.^14^ GC analysis identifies the causal relationships between pairs of electrograms recorded from different locations within a cardiac chamber. The degree and pattern of causal relationships of electrogram signals within the atrium provide indirect information about dominant propagation patterns in AF and the AF electrophenotype, as illustrated in Figure 1. For each electrogram signal, GC analysis used a linear model to predict the future sample values from given past values from the nearest nodes within a neighborhood radius of 10 mm, including the node itself. To ensure the robustness of the model, weights were optimized using lasso regression, which enforces sparsity by retaining only a few nonzero weights. This approach enhances the reliability of connections identified, focusing on dominant activation patterns while minimizing spurious connections. Given the nonstationary nature of AF signals, applying GC over longer signal recording durations only reveals stationary connections. The use of high-resolution, simultaneous noncontact mapping via the AcQMap system further ensured accurate characterization of AF dynamics by minimizing the impact of random noise, artifacts, and interpolation errors. These methodological approaches align with established best practices in electrophysiological research, allowing for a detailed and precise analysis of AF propagation dynamics. By combining these tools and techniques, the study provides a robust framework for understanding the mechanistic underpinnings of AF in a patient-specific manner.

### Parameters to quantify connectivity

We applied 3 parameters computed using causal connections obtained from conditional GC^20^ to describe the fibrillation dynamics in PsAF.

#### Causality pairing index

Causality pairing index (CPI) is a measure of the degree of connectivity/organization of the AF. It is computed as a ratio of the total number of connections found to the total possible connections. It ranges from 0 to 1, where 0 means no connections found and 1 means every node is connected to every other node. An extreme case of a macroreentrant tachycardia would be expected to have a CPI close to 1 given that the activation at an electrode site can be predicted by an adjacent electrode, given the organized nature of this rhythm. More “organized” AF would be expected to have greater CPI than “disorganized” AF. Further details are presented in Supplemental Methods and Supplemental Figure 1.

#### Directional dispersion

Directional dispersion (DD) characterizes “smoothness” of flow,^21^^,^^22^ analogous to conduction vector dispersion, with highly heterogeneous conduction having a high DD. For each signal, DD computes the direction of flow and its similarity with the direction of neighboring nodes. DD ranges from 0 and 1, where 0 means the direction of the given node is the same as neighboring nodes and 1 means it is the 180° opposite direction. The calculation of DD is described in detail in the Supplemental Methods and Supplemental Figure 2.

#### Net outflow

Net outflow for a region is computed as total connections going out from the region subtracted from the total connections coming in.^23^ This is normalized by the total possible connections between the region and outside. Net outflow can discern the direction of connections between regions. A positive value of net outflow for a region indicates the region as a source of information flow (which may be a region of an AF driver), whereas a negative value reflects its characteristics to be a sink. It ranges from −1 to 1, where −1 indicates all the possible connections from outside of regions are coming in. Further details are described in Supplemental Methods.

## Results

A total of 21 participants, with an average age of 64 years and 33% women, were included in the study. Patient characteristics are presented in Table 1. Patients were in PsAF for an average of 9.4 months on 2 antiarrhythmic drugs before ablation, with a mean CHAD_2_S_2_VASc of 2. The average LA dimension was 43.1 mm, with moderate mitral regurgitation in 24% of participants and an average ejection fraction of 49%. Ultimately, 65% of participants were free from AF at 11 months of follow-up.

**Table 1:** Participant characteristics

Variable	N = 21
Age, y	63.8 (11.1)
Women, %	7 (33%)
Persistent atrial fibrillation, %	21 (100%)
Months since diagnosis	9.4 (5.2)
Heart rate	81.4 (13.8)
Systolic blood pressure	127.0 (17.9)
Diastolic blood pressure	77.0 (11.9)
Body mass index	27.6 (3.7)
Hypertension	9 (43%)
Type 2 diabetes mellitus	4 (19%)
Heart failure	8 (40%)
Stroke	1 (5%)
Chronic kidney disease	0 (0%)
Ex-smoker	9 (43%)
Antiarrhythmic drugs (count)	2 (1)
CHAD_2_S_2_VASc	2 (1)
LA diameter, mm	43.1 (5.0)
Moderate mitral regurgitation	5 (24%)
LVEF, %	49.2 (13.4)
Follow-up in months	11 (5)
Freedom from AF at follow-up	65%

Values are presented as mean (standard deviation) and number (percentage).

LA = left atrium; LVEF = left ventricular ejection fraction.

**Figure 2:**
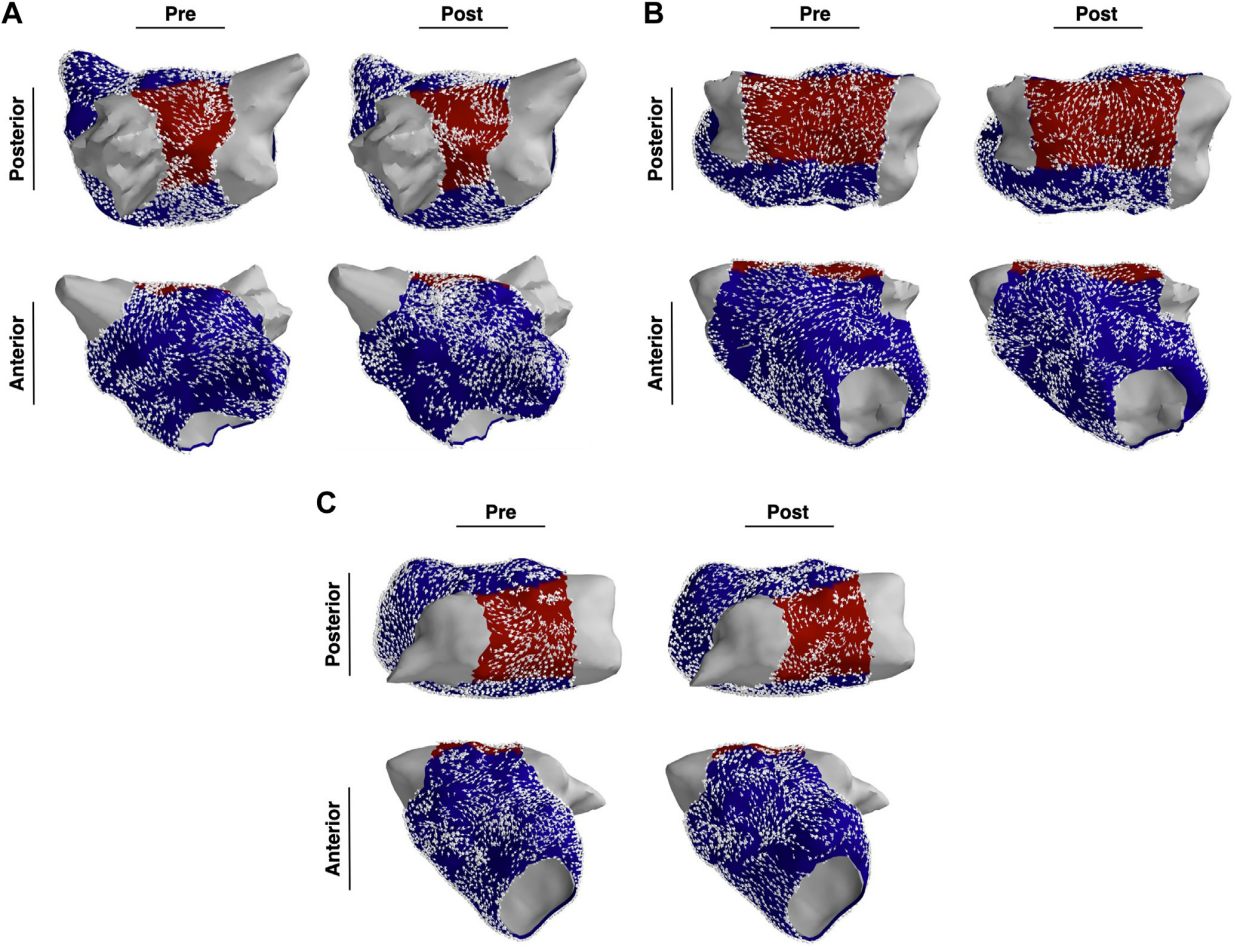
Three representative examples showing left atrial Granger causality connectivity maps. There is no significant difference in connectivity by region or before/after pulmonary vein isolation. Top panels show posterior view; bottom panels show an anterior view. The pulmonary veins are depicted in gray, the posterior wall is depicted in *red*, and the rest of the left atrium is *blue*.

Noncontact mapping was performed in patients undergoing ablation for PsAF. Mapping was performed before and after PVI. The first 5 seconds of AF data was used to compute connectivity measures before and after PVI. The LA was divided into 2 regions, the PW and the rest of the LA (Figure 2).

### CPI

**Figure 3:**
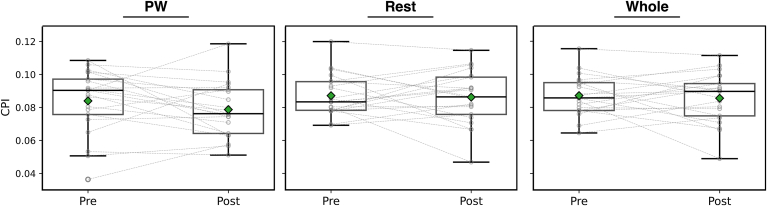
CPI was computed before and after pulmonary vein isolation for the left atrium as a whole and by region. There were no significant differences in CPI by region or before/after PVI. CPI = causality pairing index; PVI = pulmonary vein isolation; PW = posterior wall.

CPI is a measure of organization, with a high CPI indicating more organized fibrillation. Examples of GC connectivity maps before and after PVI are presented in Figure 3. CPI did not significantly change overall after PVI (0.087 ± 0.012 vs 0.086 ± 0.015; *P* = .64) or by region (PW, 0.084 ± 0.020 vs 0.079 ± 0.017; *P* = .20; rest, 0.087 ± 0.013 vs 0.086 ± 0.016; *P* = .80) (Figure 3).

### DD

**Figure 4:**
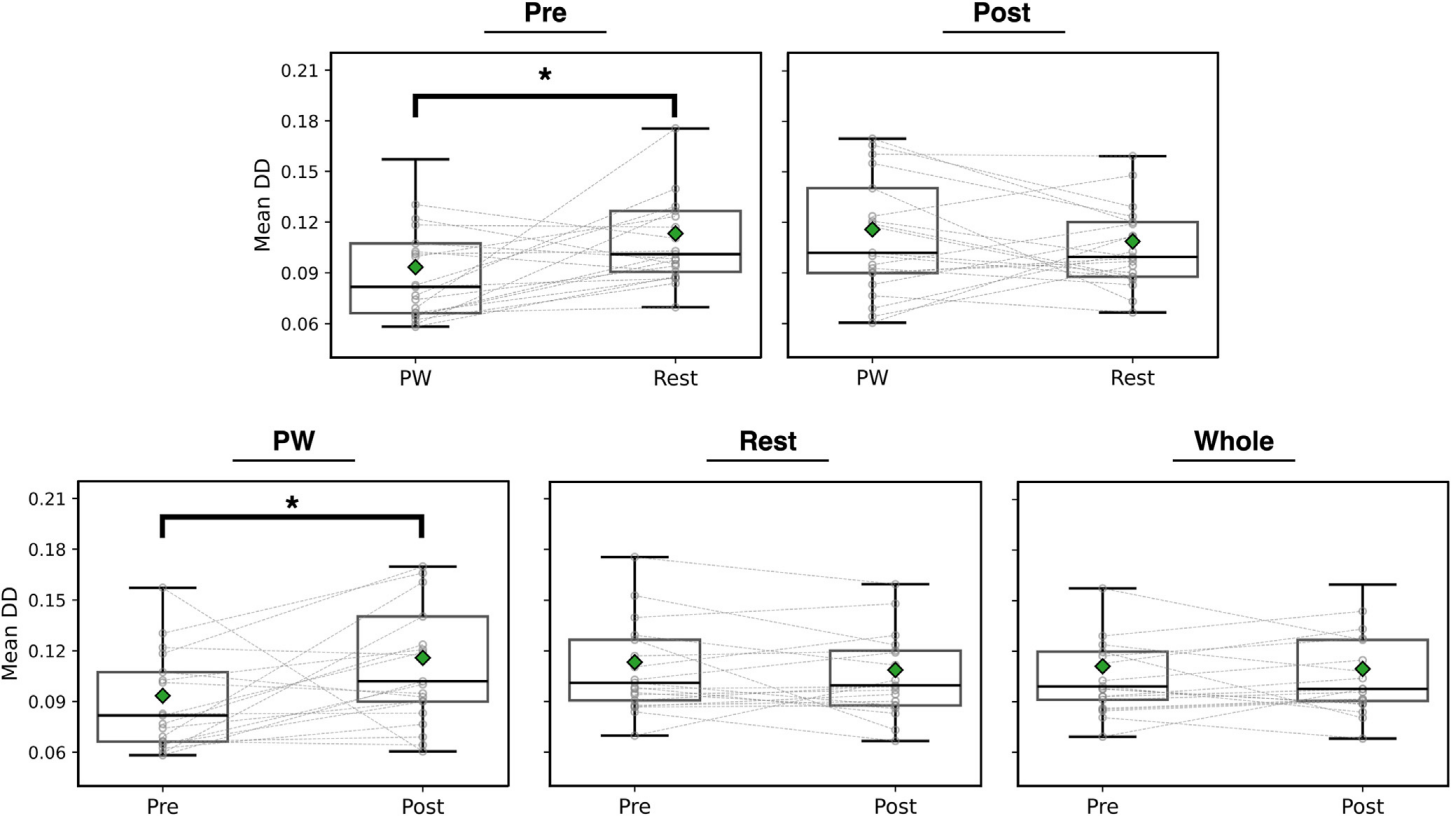
Mean DD is a measure of conduction heterogeneity. Mean DD was significantly lower in PW than the rest of the left atrium before pulmonary vein isolation, but not after ablation. Overall, mean DD in the PW significantly increased after pulmonary vein isolation; however, there were a minority of cases where the opposite trend is observed. DD = directional dispersion; PW = posterior wall.

**Figure 5:**
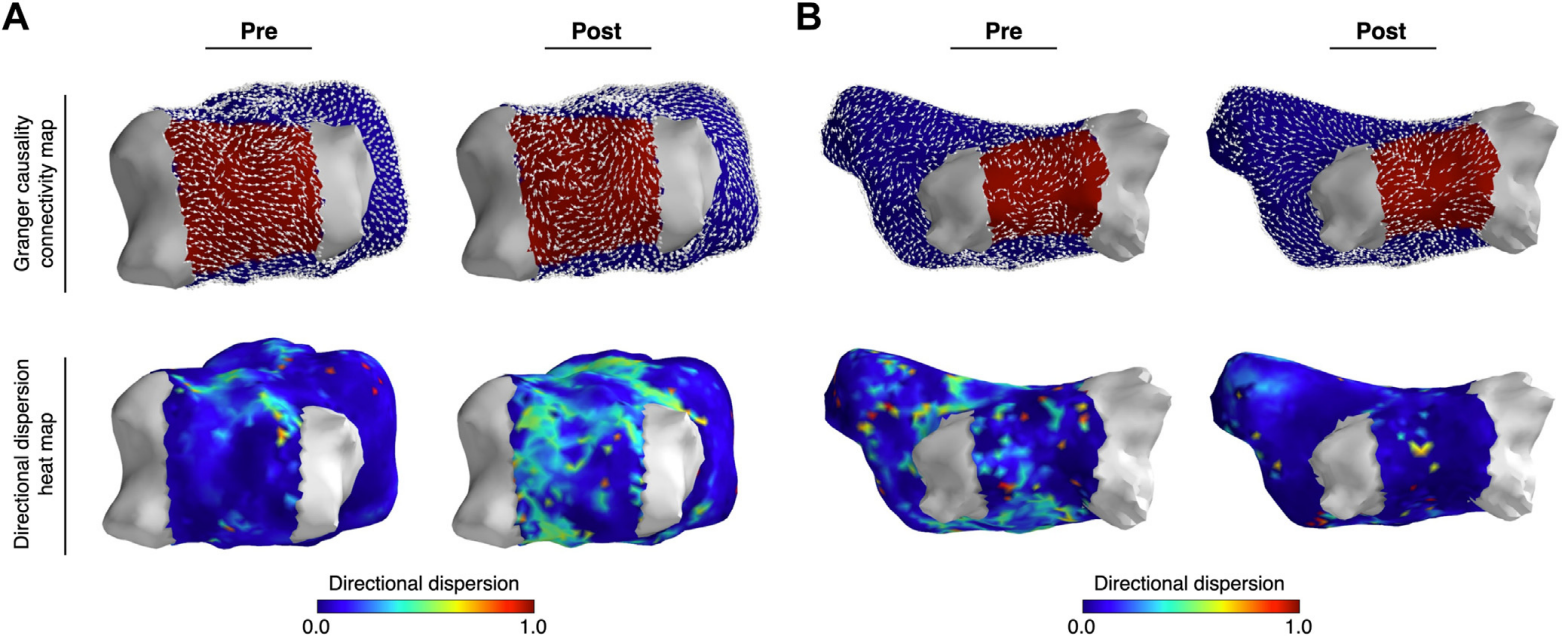
Representative examples showing Granger causality connectivity maps and directional dispersion heat maps. **A:** Directional dispersion in the posterior wall (PW) increases after pulmonary vein isolation. **B:** Directional dispersion in the PW decreases after pulmonary vein isolation. The pulmonary veins are depicted in gray; the PW is depicted in red, and the rest of the left atrium is blue.

DD quantifies conduction heterogeneity in each area of interest; higher DD signifies greater conduction heterogeneity. Before PVI, mean DD was lower in the PW than the rest of the LA (Figure 3) (0.093 ± 0.036 vs 0.11 ± 0.043; *P* = .017). PW DD increased after PVI (Figure 4) (0.093 ± 0.036 vs 0.12 ± 0.043; *P* = .045), whereas there was no change in the rest of the LA (0.11 ± 0.034 vs 0.11 ± 0.030; *P* = .52). Examples of low to high and high to low DD in the PW are presented in Figure 5.

### Net outflow for the LA PW

**Figure 6:**
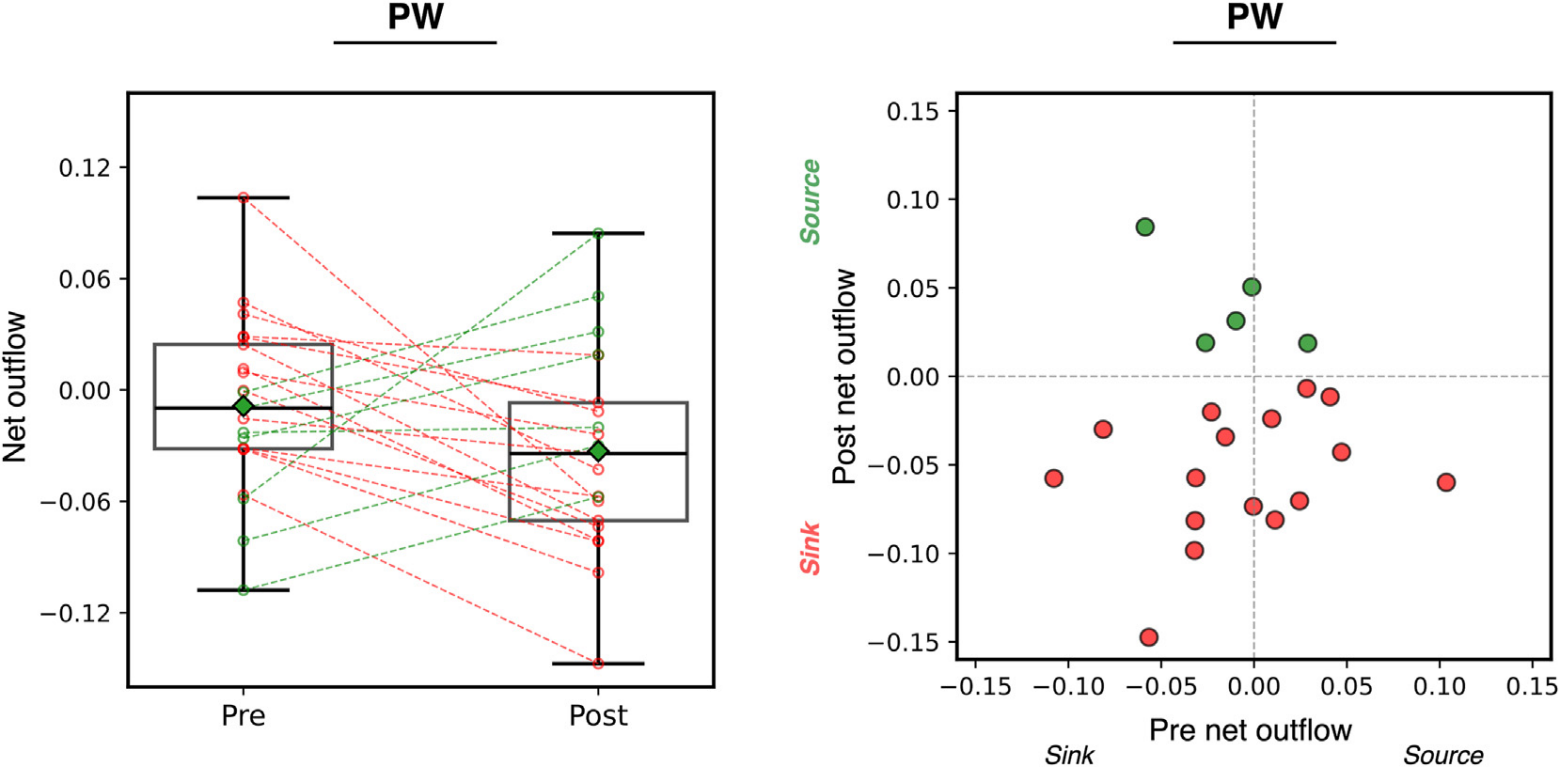
Granger causality connectivity maps were used to compute net outflow from PW. Positive net outflow indicates the PW was a net source, whereas negative indicates a net sink. Overall, there was no significant change in outflow after pulmonary vein isolation. After pulmonary vein isolation, in the majority the PW was a net sink, but was a source in a minority of cases. PW = posterior wall.

**Figure 7:**
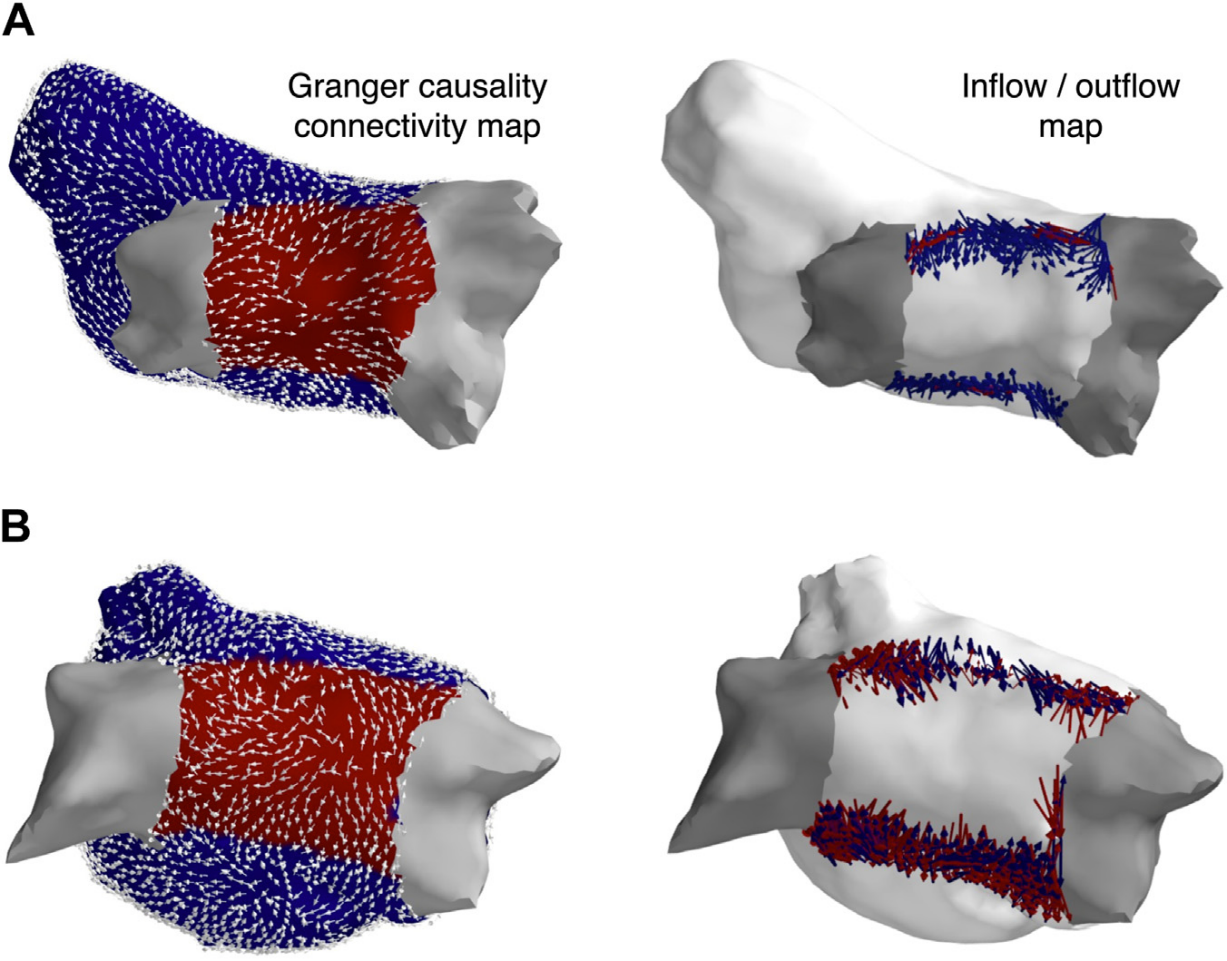
Representative examples showing Granger causality connectivity maps and posterior wall outflow after pulmonary vein isolation. In the inflow/outflow maps, *blue arrows* indicate inflow into the posterior wall and *red arrows* indicate outflow. **A:** Example of a net sink. **B:** Example of a new source. The pulmonary veins are depicted in *gray*, the posterior wall is depicted in *red*, and the rest of the left atrium is *blue*.

To understand the significance of the PW as a potential region that may harbor AF drivers after PVI, we computed the net outflow, which describes the overall direction of connections between regions. We found the net outflow of the PW was, on average, zero (ie, equal connections going into and out of the PW) before PVI (−0.0086 ± 0.047, null hypothesis − net outflow ≈ 0, 1-sample *t* test; *P* = .41). However, after PVI, the net outflow was, on average, negative (after PVI, −0.033 ± 0.054; *P* = 0.011), suggesting that in most cases the PW becomes a net sink after PVI. Figure 6 shows the PW in 16 samples was a net sink after PVI; 5 were a net source. Figure 7 shows representative examples where the PW is a net sink and net source.

## Discussion

In this study, we demonstrate the feasibility of GC to quantify global fibrillation organization, characterize dominant propagation patterns, and identify driver regions (sources) and sinks in simultaneously collected noncontact charge density PsAF maps. We found that, overall, the degree of organization does not change after PVI but that PWI is likely ineffective as an empiric adjunctive procedure because the PW becomes a net sink for atrial activity after PVI, on average.

### GC applied to noncontact charge density maps

We have previously described and validated fibrillation analysis using GC-based methods in conjunction with optical mapping and sequentially acquired clinical data.^14^ A major limitation of sequential data collection is the inherently chaotic and dynamic nature of AF, which precludes the “stitching” of regional data collected in sequence and nonsimultaneously. Here, we present a global, simultaneous approach, which provides a more accurate representation of AF dynamics.

Our results demonstrate that global AF organization remains largely unchanged after PVI, contrasting with previous studies that have reported a prolongation of the AF cycle length and a reduction in complex fractionated electrograms, suggesting increased AF organization after PVI.^2^^,^^3^^,^^24^^,^^25^ However, we observed notable alterations in the fibrillation dynamics of the PW, specifically an increase in conduction heterogeneity after PVI. This increase in heterogeneity in the PW may be caused by PVI reducing the critical mass of the PW,^26^ which could disrupt potential driver regions responsible for laminar flow with low conduction heterogeneity. These findings challenge the conventional understanding of post-PVI AF behavior.

### PW dynamics

Our findings provide insight into the null results observed in the CAPLA trials with PWI for PsAF ablation.^8^^,^^10^ We identified that only a minority of patients exhibit net positive outflow from the PW (ie, the PW acts as a source) after PVI. On average, the PW functioned as a net sink, suggesting that electrical isolation of this region may offer limited benefit for most patients. In line with the critical mass hypothesis,^26^ PVI resulting in a reduction in the effective PW area, compartmentalizing the region and diminishing net outflow, could explain our findings.

However, it should be noted that, in 24% of patients, the posterior became a net source after PVI, highlighting the heterogeneous response to PVI. Our results suggest that this smaller group of patients may benefit from PWI and that adjunctive strategies should be personalized and more targeted to patients most likely to benefit from the additional lesions, rather than be performed in all-comers in a one-size-fits-all manner.

Although this study primarily focused on AF dynamics, the observed increase in DD within the PW after PVI may offer indirect insights into the development of post-PVI atrial flutter. The increase in conduction heterogeneity could predispose to the formation of re-entrant circuits, potentially contributing to atrial flutter. This hypothesis aligns with clinical observations of atrial flutter after PVI and requires further exploration.^27^

### Limitations

This study has several limitations that warrant consideration. First, the small sample size limits the generalizability of the findings across diverse patient populations. In addition, the single-center nature of the study may introduce center-specific biases in patient selection, procedural techniques, and outcomes, which could reduce the external validity of our results. Importantly, this study represents the first attempt to apply GC analysis to atrial electrograms obtained from noncontact mapping during myocardial contraction. Although GC is a robust statistical method for identifying directional dependencies in time series data, its application in this setting introduces novel challenges owing to inherent uncertainties in the virtual signal reconstruction based on incompletely validated dipole density models, such as those underpinning the AcQMap system, when applied to complex fibrillation dynamics reconstructed from a small number of noncontact sensors potentially resulting in some degree of signal fusion or spatial ambiguity. Moreover, GC itself relies on linear and stationary assumptions, which may not fully capture the complex, nonlinear dynamics of AF. Indeed, GC may reflect not only the true directional influence of wavefront propagation but also underlying local spatial correlation patterns, particularly in the setting of complex and heterogeneous conduction. Although our previous work and others have used GC as a surrogate marker of driver activity, we acknowledge that spatial proximity and shared activation dynamics may also contribute to observed GC relationships.^28^ Although we mitigated potential inaccuracies by applying strict statistical thresholds and optimizing weights using lasso regression to minimize spurious connections, further validation using nonlinear, spatially decorrelated models might enhance robustness and provide a more comprehensive understanding of AF behavior. The interpretation of PW net flow as indicative of its role as a source or sink in AF propagation is exploratory and based on mechanistic insights derived from GC analysis. Although our findings align with previous studies highlighting the PW’s importance, direct evidence linking net flow metrics to arrhythmogenic behavior and long-term clinical outcomes data, including freedom from AF stratified by net flow directionality, constrains the immediate clinical applicability of our findings. Finally, the study was limited to PVI alone, without the assessment of adjunctive ablation strategies, which might have provided further insights into PW dynamics and improved our understanding of their mechanistic significance in AF management.

### Clinical relevance of findings

Our findings suggest that empiric isolation of the PW after PVI is not warranted, given that the PW may no longer be an important net source of drivers. The ability of GC to characterize the PW as a net sink or source on an individualized basis immediately after PVI positions it as a valuable clinical decision-making tool, potentially guiding real-time recommendations for adjunctive PWI in the catheterization laboratory for the minority of patients who might benefit. Although the current models are not optimized for real-time processing, future implementations could leverage advancements in open-source real-time signal processing software packages.^29^ GC or other connectivity mapping may also be able to guide ablation in other areas, including driver ablation; however, this requires further evaluation. Future investigations should evaluate the clinical utility of GC-derived metrics, such as net flow and DD, in predicting outcomes after PVI, including the development of atrial flutter and freedom from AF. In addition, the relationship between these metrics and established electrophysiological markers, such as fractionated electrograms and rotors, should be explored to better understand their potential role in guiding personalized ablation strategies.

### Conclusion

In this study, we describe for the first time the application of GC to global, simultaneous AF mapping data. We found that on average the PW is a net sink after PVI, and therefore, PWI will not be beneficial in most patients, providing mechanistic insight into the null randomized controlled trials for PWI. GC is positioned as a valuable clinical decision tool to select patients who may benefit from PWI to guide personalized ablation strategies in PsAF.

#### Perspectives

##### Competency in medical knowledge

We demonstrate the application of GC analysis to AF dynamics suggesting that the PW becomes a net sink after PVI, although there is a minority that develops a source and therefore might benefit from personalized PWI strategies.

##### Translational outlook

The results indicate mechanistic insight as to why empiric PWI does not significantly improve AF outcomes in most patients, aligning with null findings from randomized trials. GC analysis emerges as a promising tool to refine patient selection, potentially enhancing the efficacy of AF ablation in a subset of individuals. Future randomized controlled trials incorporating patient-specific GC analysis are essential to evaluate this targeted approach.
