# Supplementary material for: Granger causality connectivity analysis of persistent atrial fibrillation dynamics reveals posterior wall mechanistic insights
Source: Heart Rhythm O2. 2025 May 11;6(8):1097–105. doi: 10.1016/j.hroo.2025.05.002 (PMC12411957; doi:10.1016/j.hroo.2025.05.002)
Supplement: Supplementary Material [file mmc1.docx]

# Granger causality connectivity analysis of persistent atrial fibrillation electro-anatomical noncontact mapping reveals insights into the posterior wall: *Supplemental Material*

**Detailed Methods**

**Causality pairing index**

Causality Pairing Index (CPI) is a measure of the degree of connectivity/organisation of the AF. CPI for entire tissue is number of nodes divided by total possible connections, which is $N^{2}-N$ (excluding self-connections). While computing CPI for a region, only connections from nodes of same region to itself are considered, i.e., connections from region A to B or A to C are not considered for computing CPI for region A. For region A, CPI would be total black dots in top left box (A-to-A) in **Supplemental Figure 1** divided by $N_{1}^{2}-N_{1}$. Similarly for region B and C, only black dots within B-to-B box (in middle) and C-to-C box (bottom right) are considered, respectively. From **Supplemental Figure 1**, it can be seen, that region C is very well self-connected, however region A and B are not so well connected.

**Directional dispersion**

Directional Dispersion (DD) characterises ‘smoothness’ of flow. Left panel of **Supplemental Figure 2** shows the computation of DD for a given node. Consider a node P, and its direction vector with unit length as $\hat{P}$. All the neighbouring nodes are $q_{i}$ with their direction vector with unit length as $\hat{q_{i}}$. DD for node P is a median of Cosine distance (*CosineDist*) of P and all the neighbouring nodes q. Cosine distance here ranges from 0 to 1 and $\hat{P}\cdot\hat{q_{i}}$ is a dot product of vectors. Considering a median here is robust against outliers. For atria, DD for each node in atria is computed with radius r = 5mm as neighbourhood. A typical distribution of DD for atria or small region was found highly skewed to 0, so to summarise DD for different regions, mainly two statistics are computed. First is mean of DD values (DD mean) and second is skewness of the distribution (DD skew). The right panel of **Supplemental Figure 2** shows example of DD for four different patches, with background colour as DD for respective connection arrow. Top left shows all the connection arrows are almost in same direction hence the DD values for all the nodes are close to 0 (blue), which indicates a low dispersion in the direction of flow. Top right shows one arrow in middle is almost 90-degree to its neighbourhood, and a DD value close to 0.5 with yellow background can be seen. Similarly, in patch, bottom left, one arrow with more than 90-degree of divergence exists. Bottom right shows the directions of arrows seem random and chaotic, which is reflected by respective heterogenous colour values of DD.

**Net outflow**

Net outflow for a region is computed as total connections going-out from region subtracted from total connections coming-in. **Supplemental Figure 1**, region B is not self-connected, however, there are many connections exist from region B to C, which makes region B as source and region C as sink.

**Supplemental Figures and Figure Legends**


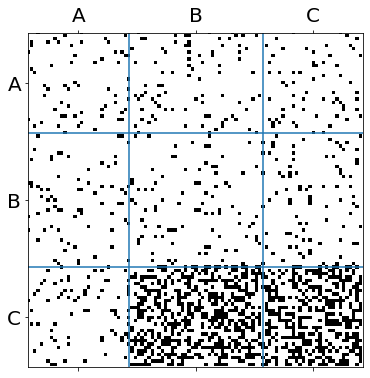


*Supplemental Figure 1: Connection matrix obtained by Granger Causality analysis*


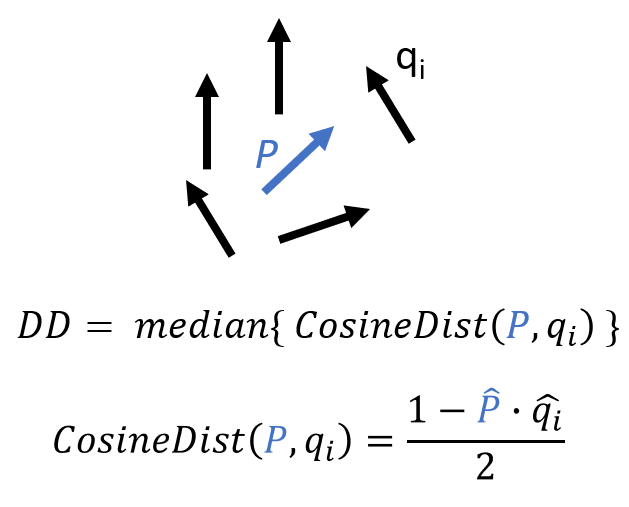


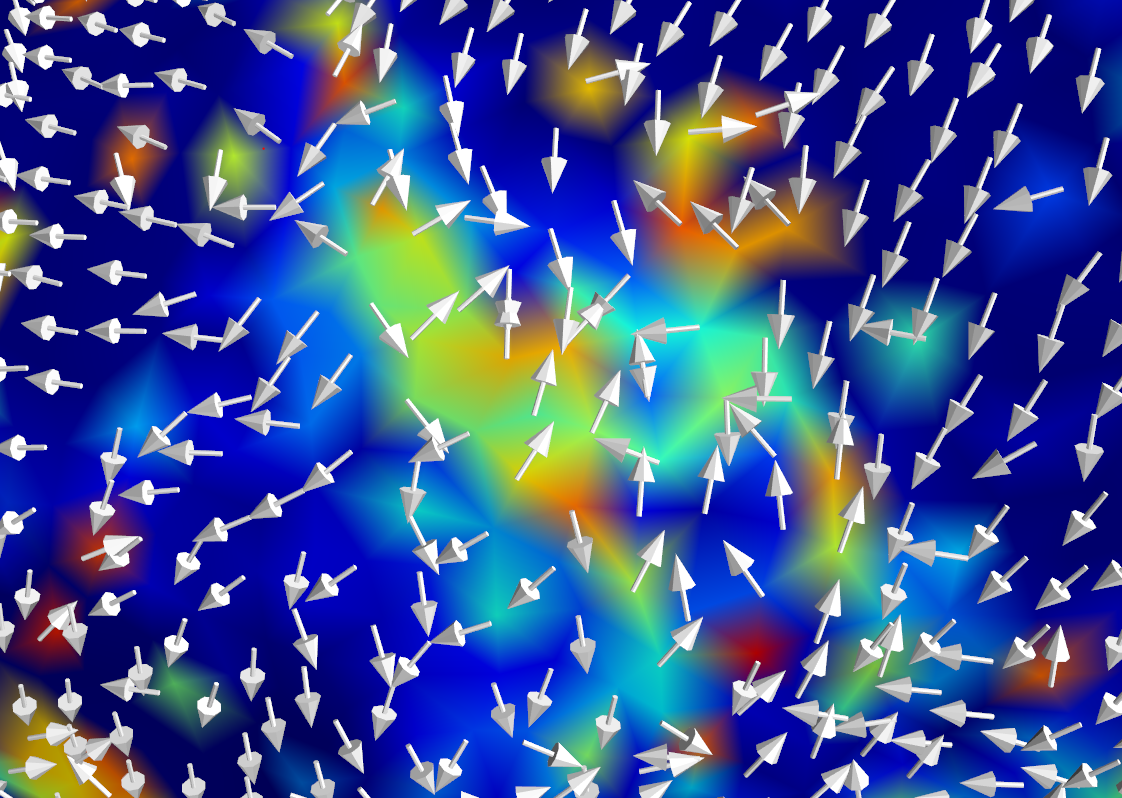

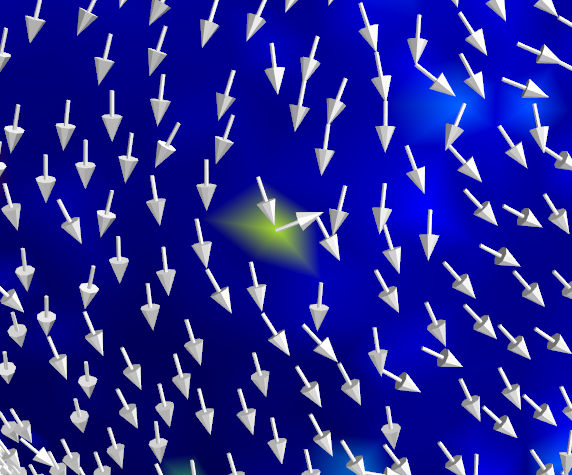

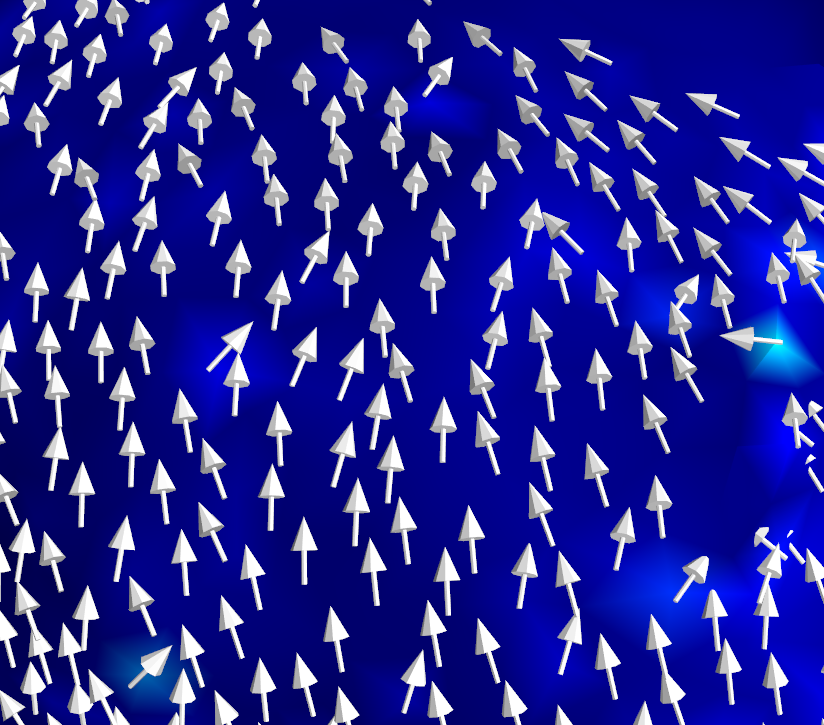

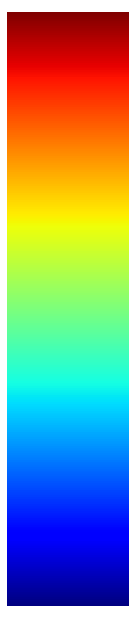


0

1

Directional Dispersion


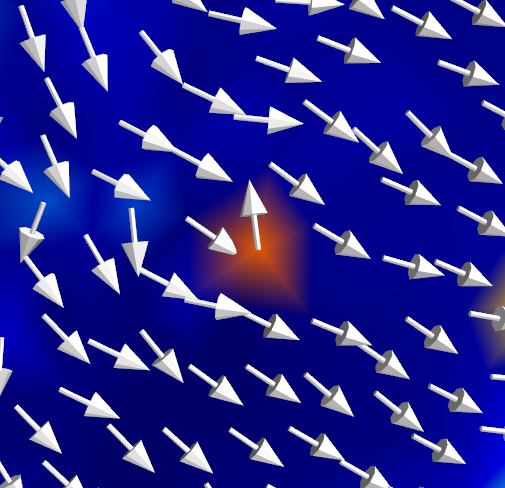


*Supplemental Figure 2: Directional Dispersion (DD). Left, computation of DD for a node P given neighbouring nodes. Right, example of four different atrial patches with DD distributions.*

*Supplement Table 1: Average Dominant Frequency (DF), Cycle Length (CL), net outflow, CPI and Average Directional Dispersion (DD Mean) of PW, before and after PVI.*

| **PIDD** | **Pre** | | | | | **Post** | | | | |
| --- | --- | --- | --- | --- | --- | --- | --- | --- | --- | --- |
|  | **DF** | **CL** | **Outflow** | **CPI** | **DD Mean** | **DF** | **CL** | **Outflow** | **CPI** | **DD Mean** |
| P1 | 12.21 | 149.09 | 0.029 | 0.073 | 0.083 | 12.24 | 150.71 | 0.019 | 0.074 | 0.083 |
| P2 | 11.88 | 143.89 | 0.041 | 0.096 | 0.195 | 12.18 | 151.48 | -0.012 | 0.074 | 0.155 |
| P3 | 12.17 | 148.92 | -0.026 | 0.036 | 0.077 | 12.04 | 147.98 | 0.019 | 0.058 | 0.124 |
| P4 | 12.20 | 148.49 | -0.108 | 0.103 | 0.101 | 11.96 | 168.06 | -0.058 | 0.085 | 0.095 |
| P5 | 12.04 | 147.66 | -0.032 | 0.051 | 0.060 | 12.08 | 151.04 | -0.082 | 0.057 | 0.140 |
| P6 | 11.98 | 143.64 | -0.059 | 0.092 | 0.065 | 12.04 | 150.74 | 0.084 | 0.092 | 0.090 |
| P7 | 11.91 | 153.42 | 0.104 | 0.079 | 0.074 | 12.03 | 173.25 | -0.060 | 0.091 | 0.090 |
| P8 | 11.87 | 175.03 | 0.011 | 0.076 | 0.130 | 11.92 | 175.77 | -0.081 | 0.063 | 0.166 |
| P9 | 12.02 | 138.30 | 0.000 | 0.086 | 0.122 | 12.13 | 146.36 | -0.074 | 0.078 | 0.117 |
| P10 | 12.17 | 165.36 | 0.024 | 0.091 | 0.082 | 12.06 | 186.91 | -0.070 | 0.080 | 0.119 |
| P11 | 12.18 | 205.45 | -0.001 | 0.109 | 0.062 | 12.87 | 166.69 | 0.051 | 0.063 | 0.077 |
| P12 | 12.14 | 137.73 | 0.047 | 0.102 | 0.118 | 12.25 | 129.05 | -0.043 | 0.076 | 0.170 |
| P13 | 11.92 | 161.32 | -0.032 | 0.106 | 0.065 | 12.17 | 169.31 | -0.098 | 0.102 | 0.100 |
| P14 | 12.15 | 143.80 | -0.010 | 0.090 | 0.067 | 11.99 | 149.62 | 0.031 | 0.119 | 0.064 |
| P15 | 11.98 | 182.97 | -0.023 | 0.088 | 0.100 | 12.78 | 188.26 | -0.020 | 0.064 | 0.236 |
| P16 | 14.19 | 144.96 | -0.057 | 0.053 | 0.157 | 11.16 | 194.41 | -0.147 | 0.051 | 0.061 |
| P17 | 11.85 | 149.35 | 0.028 | 0.090 | 0.103 | 12.00 | 153.90 | -0.007 | 0.076 | 0.121 |
| P18 | 12.77 | 163.61 | -0.031 | 0.065 | 0.058 | 12.52 | 165.06 | -0.057 | 0.094 | 0.102 |
| P19 | 12.03 | 156.93 | -0.081 | 0.097 | 0.107 | 12.30 | 149.50 | -0.030 | 0.090 | 0.093 |
| P20 | 11.96 | 174.08 | 0.010 | 0.081 | 0.069 | 12.06 | 197.23 | -0.024 | 0.071 | 0.161 |
| P21 | 12.18 | 183.14 | -0.015 | 0.101 | 0.066 | 12.22 | 180.79 | -0.034 | 0.095 | 0.069 |
| **Mean** | **12.18** | **157.96** | **-0.01** | **0.08** | **0.09** | **12.14** | **164.10** | **-0.03** | **0.08** | **0.12** |
| **SD** | 0.50 | 17.58 | 0.047 | 0.019 | 0.036 | 0.34 | 18.29 | 0.054 | 0.017 | 0.043 |
| **SE** | 0.11 | 3.93 | 0.011 | 0.004 | 0.008 | 0.08 | 4.09 | 0.012 | 0.004 | 0.010 |

Visualising DF and CL with outflow (S. Figure 3 and S. Figure 4) reveals no specific association.


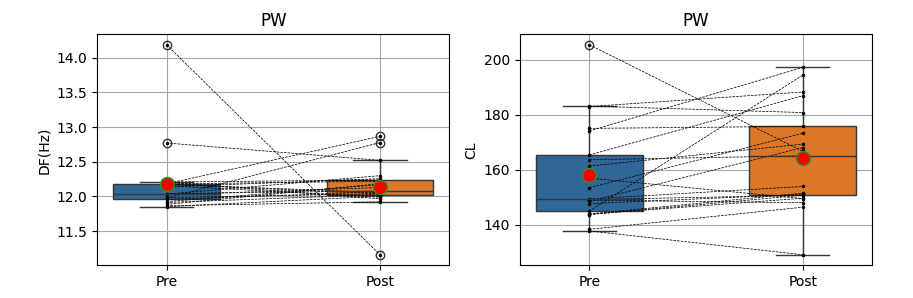


*Supplement Figure 3: Pre and Post PVI for PW (a) DF (b) CL*


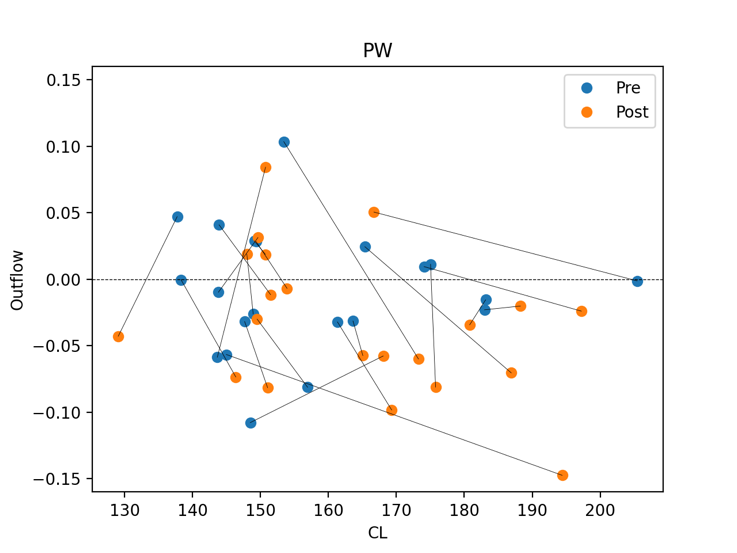

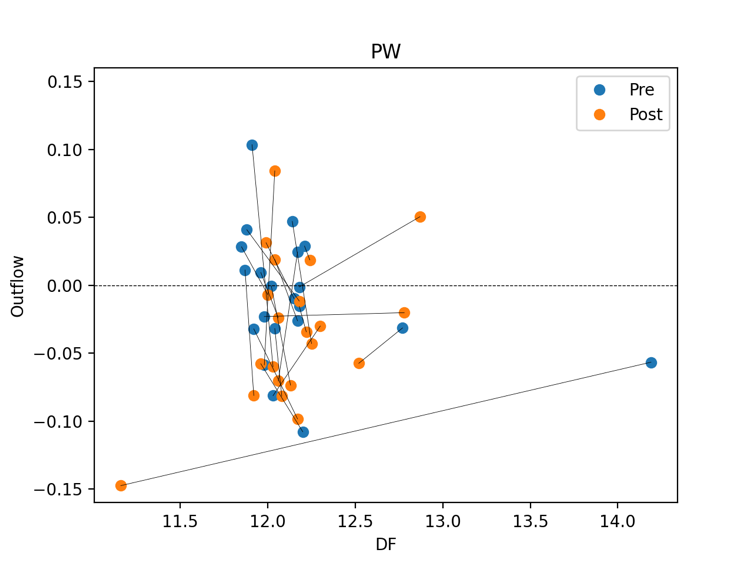


*Supplement Figure 4: (a) DF vs Outflow (b) CL vs outflow.*

Supplement Table 2: Change in Average Dominant Frequency (DF), Cycle Length (CL), net outflow, CPI and Average Directional Dispersion (DD Mean) of PW, before and after PVI.

| **PIDD** | **Change (Post – Pre)** | | | | |
| --- | --- | --- | --- | --- | --- |
|  | **DF** | **CL** | **Outflow** | **CPI** | **DD Mean** |
| P1 | 0.03 | 1.61 | -0.01 | 0.00 | 0.00 |
| P2 | 0.30 | 7.59 | -0.05 | -0.02 | -0.04 |
| P3 | -0.14 | -0.94 | 0.05 | 0.02 | 0.05 |
| P4 | -0.24 | 19.58 | 0.05 | -0.02 | -0.01 |
| P5 | 0.04 | 3.38 | -0.05 | 0.01 | 0.08 |
| P6 | 0.05 | 7.10 | 0.14 | 0.00 | 0.03 |
| P7 | 0.11 | 19.83 | -0.16 | 0.01 | 0.02 |
| P8 | 0.06 | 0.74 | -0.09 | -0.01 | 0.04 |
| P9 | 0.11 | 8.06 | -0.07 | -0.01 | 0.00 |
| P10 | -0.12 | 21.54 | -0.09 | -0.01 | 0.04 |
| P11 | 0.69 | -38.76 | 0.05 | -0.05 | 0.01 |
| P12 | 0.12 | -8.68 | -0.09 | -0.03 | 0.05 |
| P13 | 0.25 | 7.99 | -0.07 | 0.00 | 0.04 |
| P14 | -0.17 | 5.82 | 0.04 | 0.03 | 0.00 |
| P15 | 0.80 | 5.29 | 0.00 | -0.02 | 0.14 |
| P16 | -3.03 | 49.45 | -0.09 | 0.00 | -0.10 |
| P17 | 0.15 | 4.55 | -0.04 | -0.01 | 0.02 |
| P18 | -0.25 | 1.45 | -0.03 | 0.03 | 0.04 |
| P19 | 0.27 | -7.43 | 0.05 | -0.01 | -0.01 |
| P20 | 0.11 | 23.15 | -0.03 | -0.01 | 0.09 |
| P21 | 0.04 | -2.35 | -0.02 | -0.01 | 0.00 |
| **Mean** | **-0.04** | **6.14** | **-0.02** | **-0.01** | **0.02** |
| **SD** | 0.733 | 16.439 | 0.070 | 0.018 | 0.048 |
| **SE** | 0.164 | 3.676 | 0.016 | 0.004 | 0.011 |
